# Editorial: Novel Immunological Biomarkers for Allogeneic HSCT Outcome

**DOI:** 10.3389/fimmu.2021.670822

**Published:** 2021-03-12

**Authors:** Raffaella Greco, Jacopo Peccatori, Francesca Bonifazi, John A. Snowden, Fabio Ciceri

**Affiliations:** ^1^ Unit of Hematology and Bone Marrow Transplantation, IRCCS San Raffaele Scientific Institute, Vita-Salute San Raffaele University, Milan, Italy; ^2^ Hematology, IRCCS Azienda Ospedaliero-Universitaria di Bologna, Bologna, Italy; ^3^ Department of Haematology, Sheffield Teaching Hospitals NHS Foundation Trust, Sheffield, United Kingdom; ^4^ Department of Oncology and Metabolism, University of Sheffield, Sheffield, United Kingdom; ^5^ University Vita-Salute San Raffaele, Milan, Italy

**Keywords:** biomarkers, allogeneic transplantation, outcomes, graft-versus-host disease, transplant-related mortality, cytokines, immunological biomarkers, proteins

Allogeneic hematopoietic stem cell transplantation (HSCT) is a curative treatment option for many malignant and non-malignant hematological disorders (1, 2). Recent improvements in the transplant procedure, better supportive care of patients, together with advances in the prevention and treatment of HSCT-related complications, have led to a decrease of transplant-related morbidity and mortality in the last decade (3). Nonetheless, HSCT is still burdened with remarkable toxicities, which have a major impact on transplant outcome, in long and short term. Leading causes of transplant-related morbidity and mortality include infections and acute and chronic graft-versus-host disease (GvHD). Moreover, the recent introduction of novel medications for disease control has further increased the occurrence of less frequent complications such as transplant-associated microangiopathy (TAM) and veno-occlusive disease (VOD).

Unfortunately, clinically based risk scores such as the standard hematopoietic cell transplantation-comorbidity index (HCT-CI) (4), often fail to identify patients who will develop the most severe complications. Thus, developing more effective strategies for the prediction and prevention of transplant complications is still an important unmet medical need. In this setting, the use of immunological biomarkers holds promise as these non-invasive and reliable laboratory tests will potentially allow to predict transplant complications before clinical signs appear, predict their peak severity before clinical progression, and even identify patients who will not respond to treatment and are at particularly high risk for subsequent morbidity and mortality. Recently, several potential immunological biomarkers have been identified in the setting of allogeneic HSCT, ranging from serum proteins and other small molecules to immune cell subsets.

In this Research Topic, we invited expert clinicians and scientists to summarize the latest advances on novel immunological biomarkers predicting comprehensive allogeneic HSCT outcomes, not only overall survival and transplant-related mortality (TRM), but also incidence of related short and long-term complications.

The Research Topic starts with the mini review from Chen and Zeiser, where the great potential of many clinically relevant biomarkers to predict the development of acute GvHD, responsiveness of affected patients to immunosuppressive treatment, risk of relapse and subsequent disease and treatment-related mortality, has been summarized. In the next article, Adom et al. have reviewed the tools used in the identification of biomarkers, defining them as diagnostic, prognostic, predictive or response to treatment variables. Moreover, their review summarizes the biomarkers currently validated for acute and chronic GvHD, and graft-versus-tumor (GVT), and the possible application of ‘omics’ technologies and new mathematical analysis, such as machine learning, to identify novel biomarkers in this setting.

The next four manuscripts explore the role of immune cell-derived biomarkers. Leotta et al. focus on the association between overall survival (OS) and plasma levels of soluble IL-2 receptor a (sIL-2Ra) and soluble extracellular domain of T cell immunoglobulin and mucin domain 3 (TIM3), while TRM was predicted by sIL-2Ra, early after HSCT procedure. They have constructed a composite scoring system able to distinguish three different groups of patients with varying rates of TRM according to the different plasma levels of these two inflammatory cytokines. Greco et al. have investigated the role of another pro-inflammatory cytokine, interleukin (IL)-6, measured baseline (before HSCT) and 7 days after allogeneic transplant using post-transplantation cyclophosphamide (PT-Cy). They validated that increased levels predicted OS, TRM and development of grade II-IV and severe acute GvHD. Recently, also the non-classical human leukocyte antigen (HLA) molecules have gained more attention in the setting of allogeneic HSCT. Kordelas et al. provided a detailed analysis on the correlation of soluble HLA-E with extended chronic GvHD and OS, independently from the most frequent HLA-E genotypes. In the subgroup analysis, this association was confirmed mainly in patients not receiving anti-thymocyte globulin (ATG). Finally, Lia et al. have reviewed the current knowledge and potential applications of extracellular vesicles (EVs) in allogeneic HSCT, in particular those derived from mesenchymal stromal cells (MSCs) and carrying immune-modulating properties. Their clinical relevance has been analyzed also in terms of potential therapeutic strategy to improve transplant outcomes.

The Research Topic continues with two manuscripts exploring the possible use of tissue injury-derived biomarkers, not directly involved in the pathogenesis of acute GvHD, but rather indicate end-organ tissue injury caused by the inflammatory processes in GvHD. Solán et al. describe their original research on suppression of tumorigenicity 2 (ST2), a member of the IL-1 receptor family, and regenerating islet-derived protein 3a (REG3a), a C-type lectin secreted by Paneth cells, as predictive biomarkers in the context of haploidentical HSCT using PT-Cy as GvHD prophylaxis. Levels of these tissue-specific proteins at day+30 after HSCT were associated with the development of acute GvHD, TRM and OS. Further exploring the context of PT-Cy based platforms, Solán et al. have validated the role of elafin as predictive biomarker of acute skin GvHD. Higher elafin plasma levels at day+15 after HSCT correlated with higher incidence of grade III-IV skin acute GvHD, providing new insights for the early identification of patients at major risk of severe skin GvHD and potentially improving treatment delivery and prognosis.

The next four articles describe the latest advances on cellular biomarkers. Parameters of long-term immune reconstitution were evaluated in a large cohort of pediatric patients by Lawitschka et al., who report a perturbation of the B-cell compartment in correlation to chronic GvHD and its clinical aspects, particularly in terms of CD19+CD27+ memory B-cells and circulating CD19+CD21^low^ B cells. Cheung et al. outline a new perspective on the use of potential mechanism-based biomarkers to predict or monitor the therapeutic effects of MSCs for the treatment of GvHD. The recent finding that apoptosis of MSC is essential for their therapeutic efficacy represents a paradigm shift in the field, reconciling previously contradictory experimental observations. In the next article, Yang et al. provide characterization of autoantibodies in patients experiencing chronic GvHD after allogeneic HSCT. Anti-nuclear (ANA), anti-Ro52 and anti-mitochondrial autoantibodies appear to be the most frequently detected autoantibodies in patients with active chronic GvHD. Following this, Sheng et al. uncover the mechanism and immunoregulatory role of NK cells in acute GvHD. Cytotoxicity of donor NK cells toward allo-reactive T cells appears central in the regulation of acute GvHD after allogeneic HSCT. The authors provide evidence that degranulation activity of NK cells is an independent risk factor for the development and severity of GvHD.

In addition, recent studies indicate that metabolic and malnutrition biomarkers might be important factors for the outcome of allogeneic HSCT, deeply interacting also with the intestinal microbiota. With reference to malnutrition, Morello et al. have conducted a systematic search trying to identify biomarkers of nutritional status potentially useful for post-transplant immune monitoring. A focus was given to citrulline, deeply connected with immune functions and useful to monitor gastrointestinal function after allogeneic HSCT. Further exploring the ‘non-classical’ biological effects and the immune-modulatory properties of vitamin D, Soto et al. reviewed the complex function in immune and cytokine regulation of vitamin D and the conflicting results when used as biomarkers for transplant outcomes and complications, such as acute and chronic GvHD.

Finally, the topic approached the impact of major complications on transplant outcomes, driving the reader into six manuscripts able to provide a detailed view on the monitoring, clinical management and immunological aspects of these transplant complications. The focus of the review by Annaloro et al. is the complex and controversial relationship between viral infections and allogeneic HSCT. Virome components potentially represent new markers of immunological recovery after HSCT. Pradier et al. have embraced the attractive idea to evaluate the quality of post-transplant immune reconstitution through the quantification of the non-pathogenic Torque Teno Virus (TTV). TTV titers at day +100 after transplant represent a potentially useful biomarker to predict complications (i.e. acute GvHD, infections) and transplant outcomes. With reference to complications involving the central nervous system and potentially related to autoimmunity, Das et al. reported a controversial case of ‘multiple sclerosis-like’ relapsing remitting encephalomyelitis following allogeneic HSCT. Sandler et al. have provided a detailed survey of EBMT centers reviewing current diagnosis and management of secondary hemophagocytic lymphohistiocytosis (sHLH) and macrophage activation syndrome (MAS), life-threatening hyperinflammatory syndromes following HSCT and CAR-T cell therapy. The topic continues with the review from Bonifazi et al. highlighting the impact of endothelial cell injury on the development of a severe transplant complication known as hepatic veno-occlusive disease (VOD) or sinusoidal obstruction syndrome (SOS). The authors describe the pathogenesis, clinical presentation, diagnostic criteria, risk factors, prophylaxis and treatment of this severe complication. Mankarious et al. explore the potential applications of biomarkers to guide individualized treatment decisions in patients affected by acute and chronic GvHD. The incorporation of GvHD biomarkers into the patient treatment pathway alongside extracorporeal photopheresis (ECP) and other specialized treatments support a proof of concept for their potential use in routine clinical management of GvHD.

In conclusion, this Research Topic collection provides a comprehensive state-of-the-art summary of the evolution of novel biomarkers in allogeneic HSCT and their implications for clinical practice. When clinically validated, biomarkers provide a powerful means of prompt and effective identification of high-risk patients at risk of major complications of HSCT, in strategies for individualized prophylaxis, pre-emptive or other early treatment interventions, thereby improving patient survival and other outcomes following allogeneic HSCT.

## Author Contributions

All authors listed have made a substantial, direct and intellectual contribution to the work, and approved it for publication.

## Conflict of Interest

The authors declare that the research was conducted in the absence of any commercial or financial relationships that could be construed as a potential conflict of interest.
